# The perceived association of oral diseases and general pathology among doctors of different specialties

**DOI:** 10.25122/jml-2022-0021

**Published:** 2022-08

**Authors:** Ioana Monica Teodorescu, Elena Preoteasa, Cristina Teodora Preoteasa, Cătălina Murariu-Măgureanu, Cristian Teodorescu

**Affiliations:** 1 Department of Complete Denture, Faculty of Dental Medicine, Carol Davila University of Medicine and Pharmacy, Bucharest, Romania; 2 Department of Scientific Research Methods-Ergonomics, Faculty of Dental Medicine, Carol Davila University of Medicine and Pharmacy, Bucharest, Romania

**Keywords:** oral pathology, general pathology, oral manifestations

## Abstract

This study aimed to highlight the perceived associations between general and oral pathology, among clinicians of different specialties. Data was collected through a questionnaire with 22 questions, with single or multiple answers, to 88 dentists, general practitioners, or pediatricians. The majority of participants were women (89.8%), aged between 30 and 39 years (52.3%), with a professional experience mostly under 10 years (61.3%). Many doctors considered that there was an association between oral and general pathologies (39.8%). The most common general pathologies associated with oral pathology were digestive disorders (n=21, 23.9%), followed by cardiovascular, genetic, endocrine and metabolic, neuropsychiatric, respiratory, hematological, immunological, and oncological pathologies. Tooth decay was mainly found in patients with digestive, respiratory, or neuropsychiatric disorders, and periodontal disease was more common in patients with obstetric disorders. Diseases of the oral mucosa, such as canker sores and gingivostomatitis, were more common in patients with digestive pathology, endocrine and metabolic, or reproductive disorders. The study pointed out that physicians, regardless of their medical training, often observed a relatively rich general pathology associated with oral conditions. It is important to differentiate between primary and secondary oral pathology, associated with general pathology and medication, establishing a correct diagnosis of the disease and treatment according to general or oral diseases and their manifestations.

## INTRODUCTION

General disorders and those of the oral cavity negatively affect the patient's well-being, quality of life, and nutritional status. Through its functions, phonation, swallowing, mastication, or esthetics, but also the role of the first portal exposed to various antigens and pathogens, the oral cavity has an essential role in the immunity and health of the whole body [1].

Numerous studies highlighted the interrelationship between the presence of oral manifestations as a consequence of either new or recurrent systemic diseases and the associated treatment [1]. The associations of general conditions with oral ones are increasingly present as people get older, and the number of comorbidities, associated medication, and oral impairment increase [2]. There is a relationship of mutual influence between oral and general health, so the presence of a suboptimal oral status will affect the diet of patients and, implicitly, their nutritional status, increasing the risk of chronic systemic disease [3].

Understanding general pathology associated with oral conditions, identified through a complex examination of the patient, is essential for all age groups and pathologies, regardless of specialty, for a correct diagnosis and consequently to reduce the risks in various medical interventions.

The study aimed to highlight the perception of general practitioners (of different specialties) and dentists regarding the general pathologies associated with the oral ones, to understand the complex human pathology, and to achieve a unitary approach.

## MATERIAL AND METHODS

This is a descriptive cross-sectional study based on a convenience sample of dentists and general physicians working in Romania recruited online in one month.

Data was collected through an online questionnaire in Romanian consisting of two parts. The questionnaire consisted of 22 questions with single or multiple answers to obtain objective data on the purpose of the research and open questions to offer participants the option of sharing their opinions.

The questionnaire was completed on the online platform Google Forms and opened for one month in May 2021. The access to complete the questionnaire was provided by distributing the link to dentists and general practitioners via email, phone messaging, or professional groups on social networks. At the beginning of the questionnaire, the participants had to agree to the informed consent to access this query.

The first part contained six questions related to demographics, specialty, seniority, and experience of doctors in the field. The second part was represented by 16 questions related to the frequency of oral pathology associated with general pathologies and the complications associated with oral pathology.

## RESULTS

The questionnaires were completed by 88 participants, dentists, or specialists in various branches of general medicine. Regarding the specialty of the doctors who participated in the study, most were general practitioners of different specialties (n=49, 55.8%), and the rest were dentists of different specialties (n=39, 44.2%). Among the general practitioners, 16 pediatricians or primary care physicians participated (18.1%), 15 resident physicians (17%), 4 primary care physicians or specialists in obstetrics-gynecology (4.5%), 3 primary care physicians or specialists in anesthesia and intensive care (3.4%), but also physicians in other specialties, such as: ophthalmology (n=1, 1.1%), diabetes, nutrition and metabolic diseases (n=2, 2.2%), infectious diseases (n=1, 1.1%), neurology (n=2, 2.2%), medical genetics (n=1, 1.1%), cardiovascular surgery (n=1, 1.1%), emergency medicine (n=1, 1.1%), psychiatry (n=2, 2.2%).

The majority of study participants were women (n=79; 89.8%), aged between 30 and 39 years (n=46, 52.3%). The study involved doctors under the age of 30 (n=13, 14.8%), between 40 and 49 years (n=20, 22.7%), 50 and 60 years (n=6, 6.8%) and 3 doctors over the age of 60 (3.4%). Regarding professional experience, most participants (61.3%) had less than 10 years of experience, and 38.7% had more than 10 years of experience. Thus, there were many participants (n=36, 40.9%) with an experience between 5 and 10 years, but also doctors with more experience, 16–20 years (n=14, 15.9%), or 11–15 years (n=10, 11.4%). 10 doctors had experience in dentistry of over 20 years (11.4%), and 20.5% had limited experience being resident doctors or having less than 5 years of expertise (n=18).

Most participants worked in urban areas (n=86, 97.7%), the preferred form of professional organization being private practice (n=36, 40.9%). Doctors from rural areas also participated in this study in a very small percentage (n=2, 2.3%), but also doctors who work in public practice (n=34, 38.6%) or who work in both public and private professional organizations (n=18, 20.5%).

The average age of patients who came most frequently at consultations was between 18 and 40 years old (n=57, 64.8%), followed by preschool children aged between 1 and 6 years (n=14, 15.9%), children aged 7–14 years (n=11, 12.5%), adolescents between 14–18 years (n=5, 5.7%) and infants between 0–1 years (n=1, 1.1%).

35 participants (39.8%) observed that oral pathology was sometimes associated with general pathology. 20 (22.7%) participants observed this association frequently, and some encountered this correlation very frequently or rarely (n=13, 14.8%). 7 doctors stated that this association was very rare (7.8%).

The most common general pathology associated with oral pathology was digestive (n=21, 23.9%), followed by cardiovascular pathology (n=15, 17%), genetic diseases (n=11, 12.5%), endocrine and metabolic disorders (n=11, 12.5%), infectious diseases (n=10, 11.4%), neuropsychiatric diseases (n=8, 9.1%), respiratory diseases (n=7, 8%), hematological diseases (n=2, 2.3%), reproductive diseases (n=1, 1.1%), immunological (n=1, 1.1%) and oncological pathology (n=1, 1.1%).

The different oral manifestations and various general disorders, according to the doctors' perception, can be observed in Table 1. The only oral manifestation found in all six categories of general conditions was dental caries, with increased frequency among all age groups. Other oral manifestations found in most of the general conditions analyzed, but with a variable frequency, were sialorrhea and xerostomia in 4 of the 6 diseases analyzed, but also erosion, candidiasis, canker sores, gingivostomatitis, observed in 3 of the 6 diseases analyzed. However, in general, doctors associated general pathologies with the presence of distinct oral manifestations. Oral manifestations encountered by at least a third of the respondents were: digestive disorders: dental caries and dental erosions, canker sores, oral candidiasis, and halitosis; respiratory diseases: dental caries and xerostomia; endocrine and metabolic disorders: dental caries; neuro-psychiatric disorders: bruxism, dental caries, generalized dental wear, motor deficits of the orofacial muscles, pronunciation difficulties; genetic disorders: dental structure abnormalities, number abnormalities, facial malformations, periodontal diseases; obstetric-gynecological diseases: periodontal manifestations, gingivostomatitis, dental caries. There were also doctors, but very few, who considered that some of the general conditions mentioned above did not associate with oral manifestations.

**Table 1:** Different oral manifestations encountered in various general ailments, according to the doctors' perception.page no 326

Oral manifestations	General disorders (n, %)					
	Digestive disorders	Respiratory disorders	Endocrine metabolic disorders	Neuropsychiatric disorders	Genetic disorders	Obstetric and gynecological disorders
**Dental caries**	49 (55.7%)	42 (47.7%)	33 (37.5%)	42 (47.7%)	29 (33%)	34 (38.6%)
**Dental erosions**	47 (53.4%)	16 (18.2%)	17 (19.3%)	25 (28.4%)	-	-
**Dental wear**	-	-	-	40 (45.5%)	-	-
**Dental structural abnormalities**	-	-	-	-	65 (73.9%)	-
**Dental anomalies of number**	-	-	-	-	49 (55.7%)	-
**Periodontal damage**	-	-	-	-	30 (34.1%)	42 (47.7%)
**Oral candidiasis**	35 (39.8%)	24 (27.3%)	20 (22.7%)	-	-	-
**Canker sores**	37 (42%)	18 (20.5%)	23 (26.1%)	-	-	17 (19.3%)
**Gingivostomatitis**	29 (33%)	11 (12.5%)	27 (30.7%)	-	-	32 (36.4%)
**Pigment spots**	-	-	1 (1.1%)	-	-	-
**Genetic keratosis**	-	-	-	-	1 (1.1%)	-
**Lichen planus buccal**	-	-	-	-	-	1 (1.1%)
**Sialorrhea**	12 (13.6%)	10 (11.4%)	9 (10.2%)	10 (11.4%)	-	7 (8%)
**Xerostomia**	12 (13.6%)	35 (39.8%)	24 (27.3%)	13 (14.8%)	-	-
**Burning sensations**	16 (18.2%)	-	-	-	-	-
**Glosodynia**	-	-	-	1 (1.1%)	-	-
**Dysgeusia**	5 (5.7%)	-	-	5 (5.7%)	-	-
**Trigeminal neuralgia**	-	-	-	13 (14.8%)	-	-
**Halitosis**	35 (39.8%)	2 (2.3%)	-	-	-	-
**Bruxism**	-	-	-	53 (60.2%)	-	-
**Orofacial motor deficiencies**	-	-	-	36 (40.9%)	-	-
**Pronunciation difficulties**	-	-	-	30 (34.1%)	-	-
**Facial malformations**	-	-	-	-	44 (50%)	-
**Absence of oral manifestations**	-	-	1 (1.1%)	1 (1.1%)	-	2 (2.3%)

"-" no responses were recorded.

The participants noticed the presence of loco-regional and general complications but also associations with some general conditions of periodontal diseases. Thus, the loco-regional and general complications of periodontal diseases observed were osteitis of the maxillary bones (n=46, 52.3%), maxillary sinusitis (n=35, 39.8%), cellulite (n=31, 35.2%), adenitis (n=26, 29.5%), cholecystitis by pyophagia in conditions of hypo- or gastric achlorhydria (n=17, 19.3%), thrombophlebitis of the cavernous sinus (n=12, 13.6%), sepsis (n=9, 10.2%), brain abscess (n=7.8%). Some doctors also mentioned the association of periodontal disease with general conditions, such as diabetes (n=1, 1.1%), rheumatoid arthritis (n=1, 1.1%) or digestive disorders (n=1, 1.1%).

Regarding the general complications associated with oral and maxillofacial infections, doctors observed the presence of localized or extended abscesses (n=62, 70.5%), bacterial endocarditis (n=49, 55.7%), cellulite or phlegmon (n=45, 51.1%), cavernous sinus thrombosis (n=35, 39.8%), brain abscess (n=26, 29.5%), mediastinitis (n=20, 22.7%) and meningitis (n=14, 15.9%).

Regarding the viral etiopathogenesis of oral diseases, doctors considered the most common association with herpes virus infection (n=79, 89.8%), but also herpangina (n=62, 70.5%), human immunodeficiency virus infection (n=59, 67%), Epstein Barr virus infection (n=47, 53.4%), Coxsackie virus (n=39, 44.3%), Human Papillomavirus infection (n=38, 43.2%), Cytomegalovirus infection (n=28, 31.8%), or hepatitis B or C virus infection (n=11, 12.5%).

Regarding the bacterial infectious pathology with manifestations in the oral cavity, the specialists observed the association of syphilis (n=73, 83%), scarlet fever (n=50, 56.8%), actinomycosis (n=33, 37.5%), tuberculosis (n=28, 31.8%), meningitis (n=5, 5.7%), Salmonella typhi infection (n=4, 4.5%), *Clostridium perfringens* infection (n=3, 3.4%) or *Escherichia coli* infection (n=1, 1.1%).

Among the digestive disorders that show manifestations in the oral cavity, the most common were gastroesophageal reflux (n=75, 85.2%), gastrointestinal candidiasis (n=55, 62.5%), Crohn's disease (n=31, 35.2%), liver disease (n=20, 22.7%), Gardner syndrome (n=13, 14.8%) and ulcerative colitis (n=13, 14.8%).

Among the respiratory diseases with manifestations in the oral cavity, the most common associations were obstructive sleep apnea (n=43, 48.9%), asthma (n=29, 33%), acute upper respiratory tract infection (n=29, 33%), cystic fibrosis (n=27, 30.7%), chronic neonatal lung disease (n=4, 4.5%).

Among the endocrine and metabolic disorders with manifestations in the oral cavity, the most common associations were diabetes (n=68, 77.3%), avitaminosis (n=68, 77.3%), anemia (n=45, 51.1%), congenital hypothyroidism (n=21, 23.9%), Cushing's syndrome (n=18, 20.5%), Addison's disease (n=18, 20.5%) and hyperparathyroidism (n=17, 19.3%).

## DISCUSSIONS

The association of general pathologies with oral ones was underlined in the specialized literature through numerous studies, which also emphasize the uncertainty of the diagnosis based only on the clinical manifestations. Thus, according to Edens, the orofacial manifestations of various systemic pathologies have similar symptoms and clinical aspects, making it very difficult to develop an exclusive diagnosis based on oral symptoms, so a complex clinical examination is required, accompanied by specialized medical evaluations and complementary explorations [4].

For any complaints in the orofacial area, a complete medical and dental examination must be performed, with an extensive evaluation, accompanied by paraclinical tests: radiological, laboratory, and biopsy. Bacterial, viral or fungal cultures can be harvested with an antibiogram or antifungal [4, 5] to obtain a correct differential diagnosis and establish a final diagnosis.

The physicians who participated in the study were dentists and general practitioners with a professional experience between 5 and 10 years of activity (n=36, 40.9%). Most participants were women (n=79, 89.8%) aged between 30 and 39 years (n=46, 52.3%). Furthermore, the majority work in urban areas (n=86, 97.7%), most frequently in private practice offices (n=36, 40.9%).

The average age of patients who come to consultations most frequently was between 18 and 40 years old (n=57, 64.8%), and preschool age between 1 and 6 years (n=14, 15.9%).

Oral pathology associated with general digestive disorders was most frequently observed in the pediatric population, with dental caries and canker sores being the predominant oral disorders. In contrast, in adults, an association of digestive and cardiovascular pathologies was observed, and the most common oral disorders were canker sores, gingivostomatitis, or dental erosions. The most frequent general pathologies perceived by dentists are cardiovascular diseases or endocrine and metabolic disorders, and the most common oral pathologies are canker sores, gingivostomatitis, or dental erosions. In digestive pathology, dentists and general physicians consider gastroesophageal reflux as the main general associated pathology, while in respiratory pathology, dentists consider obstructive sleep apnea as associated symptomatology. General practitioners associate it with acute upper respiratory tract infections. In endocrine-metabolic pathology, both diabetes and avitaminosis are correlated with both specialties.

According to the participants, oral pathology is sometimes associated with general pathology (n=35, 39.8%), and some doctors encountered this correlation either very frequently or rarely (n=13, 14.8%).

The most common general pathology associated with oral pathology was digestive pathology (n=21, 23.9%). Gastroesophageal reflux disease (n=75, 85.2%) was the digestive disorder most frequently associated with oral manifestations, such as sialorrhea, xerostomia, burning sensation, dysgeusia, halitosis, odynophagia or tooth sensitivity at different temperatures. Periodontal damage and tooth erosion are also sequelae of gastroesophageal reflux disease. With regard to liver damage, studies have shown discoloration of the mucous membranes with a yellowish aspect, especially in the area of the lingual frenulum or soft palate. Petechial areas or gingivitis may also occur. Burning mouth syndrome is often associated with diabetes mellitus and nutritional deficiencies, as well as a triad of pain, altered taste, and xerostomia [6]. Sialadenitis is most commonly associated with cirrhosis of the liver but also with malnutrition or metabolic changes [7]. Gardner's syndrome has an incidence between 1/4000 and 1/12000 and associates multiple intestinal polyps and tumors with other locations: oromaxillofacial osteomas, fibroids, squamous cysts, or thyroid cancer [8].

Cardiovascular, genetic, endocrine, and metabolic pathologies took up the following places regarding the frequency of associations between general and oral pathologies.

In cardiovascular pathology, there are interactions due to antihypertensive medication, with manifestations such as xerostomia or increases in gingival volume. Medication for heart failure causes dry mouth, taste changes, or lichenoid reactions [9, 10]. The highest prevalence of periodontal disease in correlation with cardiovascular pathologies is encountered in the third-aged population [10], with alterations of salivary flow, a condition in which oral candidiasis and prosthetic stomatitis are often reported [11]. The metabolic syndrome presents an important association with inflammatory-destructive periodontal lesions [12].

The most common oral manifestations encountered in genetic disorders were dental structure abnormalities (n=65, 73.9%) and number anomalies (n=49, 55.7%).

In respiratory pathology, obstructive sleep apnea (n=43, 48.9%) was the respiratory condition most frequently associated with oral pathology. In the literature, it is associated with craniofacial abnormalities and dental carious lesions, periodontal disease, or xerostomia [13]. Oral hygiene also plays an important role in preventing aspiration pneumonia, the oral microbial flora being associated with respiratory complications [14]. Patients that develop mouth breathing may have allergies, postural problems, or facial abnormalities [15].

Dental caries are the most frequently associated oral pathologies with general pathology, according to respondents, and develop both in diseases of the digestive tract (n=49, 55.7%), respiratory tract (n=42, 47.7%), and endocrine-metabolic diseases (n=33, 37.5%). Dental caries ranked second in neuro-psychiatric pathology (n=42, 47.7%) and the field of obstetrics-gynecology (n=34, 38.6%), with the highest percentage of total association between general and oral pathology.

In neuropsychiatric disorders, the most common oral manifestation encountered by physicians was bruxism (n=53, 60.2%). Nocturnal bruxism in children has a prevalence between 5–49.6% in the literature [16]. The presence of mental disorders could act as a trigger point for bruxism once they cause changes in the regulation of the central nervous system [17]. In neurological pathology, motor or cognitive deficits occur due to strokes or Parkinson's disease, with decreased ability to care for the oral cavity. In multiple sclerosis, trigeminal neuralgia occurs in 1.9% of cases [18]. Children with cerebral palsy have a higher incidence of dental caries; therefore, preventive measures should be taken into account [19].

Periodontal disease (n=42, 47.7%) was the most commonly associated with reproductive system pathology. Correlations have been established between periodontal disease of mothers and premature infants with low weight at birth and a direct relationship between the severity of periodontitis and the risk of premature birth [20].

The most frequent loco-regional and general complications associated with periodontal disease were osteitis and osteomyelitis of the maxillary bones (n=46, 52.3%) and maxillary sinusitis (n=35, 39.8%). Regarding the general complications associated with oro-maxillofacial infections, localized or extended infections (n=62, 70.5%) and bacterial endocarditis (n=49, 55.7%) were the most common. According to DeCroos, orbital abscesses occurred in 72.7% of cases with odontogenic orbital cellulitis, one of the complications not specified by the physicians [21].

Regarding the viral etiopathogenesis of oral diseases, physicians assessed that the herpes virus infection (n=79, 89.8%) ranks first in terms of associations. In case of viral infections with oral manifestations, the herpes simplex virus is most common. In the primary herpes infection (type 1 herpes simplex virus), there are gingival inflammations with characteristically grouped vesicles on the keratinized mucosa that converge in ulcerative areas, except for immunocompromised patients where they can also appear on the non-keratinized mucosa. Coxsackie virus is the etiology of hand-foot-mouth disease, which generally occurs in children under 10 years of age, as well as herpangina, which occurs in the posterior oropharyngeal area [7, 8]. The age groups 6 months and 3 years seem to be the most prone to herpes infection. The virus has a latency period of 2–12 days, the infection being accompanied by a fever above 38⁰C, cough, lymphadenopathy, halitosis, sialorrhea, and dehydration. According to Napenas, lichen planus was associated with hepatitis C virus infection in 0.1%–35% of cases [7].

In the case of bacterial infectious pathology with manifestations in the oral cavity, specialists observed the association of syphilis (n=73, 83%) and scarlet fever (n=50, 56.8%). Bacterial infections with manifestations in the oral cavity have a different pathological pattern compared to specific bacterial etiology of dental caries. The oral aspect of bacterial infections is represented by erosive-ulcerative mucosal lesions, sometimes covered with fibrinogen. Syphilis and tuberculosis have similar oral manifestations [18].

Among the endocrine and metabolic disorders that present manifestations in the oral cavity, the association of diabetes (n=68, 77.3%) was most frequently observed, as well as avitaminosis (n=68, 77.3%), with more frequent localization in the mandible, without patients complaining of pain [8].

Type 2 diabetes is most commonly associated with periodontal disease but also with hyposialia, sialadenitis, and more common *Candida Albicans* infections, due to high sugar levels and low salivary flow, correlated with changes in salivary pH [7]. In diabetes, there are alterations in polymorphonuclear function, distorted response to bacterial or antigen attacks, and changes in T lymphocytes [8].

Avitaminosis A and E are associated with periodontal lesions, and the deficiency of vitamins B2 (riboflavin), B3 (niacin), B6 (pyridoxine), B9 (folic acid), B12 (cobalamin) is associated with tongue atrophy with glossodynia or cheilitis of the lips. B7 (biotin) deficiency has extraoral manifestations exclusively, with erythema and scaling around the mouth, nose, and eyes. Folic acid deficiency is often implicated in periodontal damage. Cobalamin deficiency causes recurrent oral aphthosis with changes in taste sensations. Vitamin C deficiency or scurvy causes gingivitis and mucosal bruising, leading to periodontal damage with mobility and dental avulsion [7]. According to Bhalla, ascorbic acid is involved in critical processes of human physiology: collagen synthesis, fatty acid transport, neurotransmitter formation, wound healing, and immunity; it most commonly occurs in people with poor diet or psychiatric disorders: developmental deficits, autism spectrum disorder [8, 22].

Iron deficiency, together with B12 deficiency, leads to taste changes and depapillation of the tongue, being a risk factor in the appearance of pseudomembranous oral candidiasis and angular cheilitis [8].

In Plummer-Vinson syndrome, glossitis with dysphagia occurs, being a favorable factor for the appearance of oral, pharyngeal, or esophageal squamous cell carcinoma [8], and zinc deficiency has been associated with dysgeusia without having any role in the appearance of canker sores [7]. Also, most patients with Plummer-Vinson syndrome are women from Northern Europe or Scandinavia in the third decade of life. Anemia is accompanied by fatigue, shortness of breath, weakness, tachycardia, and pallor. Since it is a premalignant disease, attention should be focused to symptoms that indicate malignancy, such as odynophagia with changes in voice, nasal regurgitation, anorexia, or weight loss [8].

In Cushing's syndrome, known as hyperadrenalism, high levels of cortisol cause changes in the facial typology with a full moon face aspect, but also intraorally, with changes in the oral mucosa, at which level dental impressions are observed, especially in the buccal area, and ulcers. In Addison's disease, known as hypoadrenalism, in which cortisol secretion is very low, pigment changes such as brown spots appear in the oral cavity [7]. According to Bhalla, the incidence of new cases is about 110–140 million per year in the Western Hemisphere, and intraoral changes are an early marker of this disease, with skin manifestations occurring much later after the onset of the disease [8].

Hyperparathyroidism causes trabecular and cortical bone changes and can lead to pathological tooth mobility over time. In congenital hypothyroidism, there are changes such as macroglossia and delayed tooth eruption [7, 23]. Slight delays in tooth eruption may occur in type 0 glycogenosis [24].

In systemic lupus erythematosus, a quarter of patients have intraoral manifestations such as ulceration, keratosis, erythema, purpura, and petechiae [18].

General and oral health should be the concern of oral treatments in children [22, 25] and adults [26], especially when it involves diseases and treatments with a high level of risk.

The limitations of the study are related, on the one hand, to the relatively small group of 88 doctors interviewed but also to the relatively low age and experience of doctors, most of whom were under 10 years.

## CONCLUSIONS

The study showed that physicians, regardless of their medical training, often observed a relatively rich general pathology associated with oral conditions, both in children and adults, with small differences in the perception of pathologies depending on medical training. General conditions such as digestive, cardiovascular, genetic, respiratory, and neuropsychic disorders are associated with oral conditions, such as caries processes, dental erosions, lesions of the oral mucosa (canker sores, candidiasis), gingivostomatitis, salivary disorders (sialorrhea or xerostomia) or taste disorders (dysgeusia).

It is important to differentiate the primary oral pathology, like dental caries or periodontal disease, without general causes, from the secondary one, associated with general pathology and medication. It is also important to establish a correct diagnosis of the disease and treatment according to the general or oral diseases and their manifestations to reduce the risk of increased damage.
